# Protocol for the CONVERT trial—Concurrent ONce-daily VErsus twice-daily RadioTherapy: an international 2-arm randomised controlled trial of concurrent chemoradiotherapy comparing twice-daily and once-daily radiotherapy schedules in patients with limited stage small cell lung cancer (LS-SCLC) and good performance status

**DOI:** 10.1136/bmjopen-2015-009849

**Published:** 2016-01-20

**Authors:** Corinne Faivre-Finn, Sally Falk, Linda Ashcroft, Michelle Bewley, Paul Lorigan, Elena Wilson, Nicki Groom, Michael Snee, Pierre Fournel, Felipe Cardenal, Andrea Bezjak, Fiona Blackhall

**Affiliations:** 1 Manchester Academic Health Science Centre, Institute of Cancer Sciences, Manchester Cancer Research Centre (MCRC), The University of Manchester, Manchester, UK; 2 Radiotherapy Related Research, The Christie NHS Foundation Trust, Manchester, UK; 3 Manchester Academic Health Science Centre Trials Coordination Unit (MAHSC-CTU), The Christie NHS Foundation Trust, Manchester, UK; 4 Department of Clinical Oncology, The Christie NHS Foundation Trust, Manchester, UK; 5 Department of Medical Oncology, The Christie NHS Foundation Trust, Manchester, UK; 6 Department of Oncology, University College London Hospitals NHS Foundation Trust, London, UK; 7 Radiotherapy Trials Quality Assurance Team, Mount Vernon Hospital, Northwood, UK; 8 St James’ Institute of Oncology, Leeds, UK; 9 Institut de Cancérologie Lucien Neuwirth, Saint-Priest en Jarez, France; 10 Department of Medical Oncology, Catalan Institute of Oncology, L'Hospitalet, Barcelona, Spain; 11 Department of Radiation Oncology, Princess Margaret Hospital, Toronto, Ontario, Canada

**Keywords:** CHEMOTHERAPY

## Abstract

**Introduction:**

Concurrent ONce-daily VErsus twice-daily RadioTherapy (CONVERT) is the only multicentre, international, randomised, phase III trial open in Europe and Canada looking at optimisation of chemoradiotherapy (RT) in limited stage small cell lung cancer (LS-SCLC). Following on from the Turrisi trial of once-daily versus twice-daily (BD) concurrent chemoradiotherapy, there is a real need for a new phase III trial using modern conformal RT techniques and investigating higher once-daily radiation dose. This trial has the potential to define a new standard chemo-RT regimen for patients with LS-SCLC and good performance status.

**Methods and analysis:**

447 patients with histologically or cytologically proven diagnosis of SCLC were recruited from 74 centres in eight countries between 2008 and 2013. Patients were randomised to receive either concurrent twice-daily RT(45 Gy in 30 twice-daily fractions over 3 weeks) or concurrent once-daily RT(66 Gy in 33 once-daily fractions over 6.5 weeks) both starting on day 22 of cycle 1. Patients are followed up until death. The primary end point of the study is overall survival and secondary end points include local progression-free survival, metastasis-free survival, acute and late toxicity based on the Common Terminology Criteria for Adverse Events V.3.0, chemotherapy and RTdose intensity.

**Ethics and dissemination:**

The trial received ethical approval from NRES Committee North West—Greater Manchester Central (07/H1008/229). There is a trial steering committee, including independent members and an independent data monitoring committee. Results will be published in a peer-reviewed journal and presented at international conferences.

**Trial registration number:**

ISRCTN91927162; Pre-results.

**Strengths and limitations of this study**

- This is an adequately powered multicentre, randomised controlled trial aiming to establish a standard chemoradiotherapy regime in limited stage small cell lung cancer.
- All patients are treated with modern radiotherapy. This trial uses and reflects the important developments in modern conformal radiotherapy techniques that have taken place in the past 20 years.
- Elderly patients are not excluded. Inclusion/exclusion criteria are based on good performance status with no upper age restriction.

## Introduction

Of the 42 000 patients diagnosed with lung cancer each year in Britain, 15% will have small cell lung cancer (SCLC) and 30% of those patients will have limited stage (LS) disease that can be encompassed within a tolerable radiation therapy field. Outcome is poor even in this early stage of disease, with a median survival of 16–24 months, using current forms of treatment.^1–3^ Combining chemotherapy and thoracic radiotherapy (RT) is the standard treatment for LS SCLC). Two meta-analyses have shown that RT associated with chemotherapy improves median survival, 3-year survival rate and local control.^4^ ^5^ Subsequently, meta-analyses of trials investigating the optimal timing and sequencing of chemo-RT have shown that the best results have been reported with early concurrent thoracic RT.^6–9^ Furthermore, it has been demonstrated that twice-daily RT is superior to once-daily RT, in the landmark Turrisi study.^3^ Patients were randomised to either 45 Gy once-daily (1.8 Gy per fraction) over 5 weeks or 45 Gy given twice-daily (1.5 Gy per fraction) over 3 weeks. In both arms, RT was given concurrently starting with the first cycle of chemotherapy (cisplatin and etoposide). Twice-daily RT improved 5-year OS (26% vs 16% in the once-daily arm), reduced the risk of thoracic relapse (52% compared with 36% in the twice-daily arm) but at the cost of increased grade 3 radiation oesophagitis (defined as inability to swallow more than liquids, or to require hospitalisation). However, there were no other significant differences in acute toxicity between the two arms and no long-term oesophageal strictures were reported. Consequently, twice-daily RT concurrently with chemotherapy is accepted as a standard regime in LS-SCLC.^10^ It is, however, unclear whether the better results in the twice-daily arm are explained by the increase in the biologically equivalent dose of radiation in the twice-daily arm or by the use of altered fractionation leading to a shorter overall treatment time. Indeed, the control dose was considered to be quite low, for example, in comparison with the NCI Canada regime of 40 Gy in 15 daily fractions.^1^ Moreover, since the late 1980s, when the Turrisi trial was designed, important progress has been made in RT techniques. The RT used in any contemporary trial should be CT-planned, conformal treatment, with individual field shaping careful dose calculation using modern planning algorithms of target and organs at risk, with image guidance, correction of set-up errors and allowance made for the effects of respiratory motion on the position of the target volume. None of these were routine from 1989–1992, when the Turrisi trial was carried out. The use of three-dimensional RT/intensity modulated RT and the omission of elective nodal irradiation are likely to result in lower rates of toxicity, particularly oesophagitis. Further studies by Choi *et al*,^11^ using once-daily RT, and Komaki *et al*,^12^ using a concomitant boost technique, have suggested that doses of 70 Gy over 7 weeks and 61.8 Gy over 5 weeks, respectively, are possible, the former being delivered in five cycles of full-dose chemotherapy.

There is, therefore, a need to improve on the current survival results, and a strong rationale to compare twice-daily with a higher dose of radiation delivered once-daily.

In view of the lack of data in the literature addressing the question of the dose and fractionation for LS-SCLC, we carried out a randomised phase III trial to establish a standard chemo-RT regimen for LS-SCLC with good performance status (PS) (Concurrent ONce-daily VErsus twice-daily RadioTherapy, CONVERT). At the time the trial was being developed, in 2006–2007, there were no international trials for this group of patients, thus an opportunity existed to set up a global trial to answer this important question. The results of the trial will be crucial in determining the best international standard treatment for routine clinical use in the treatment of patients with limited-stage SCLC and good PS. In addition, the translational studies carried out in parallel with CONVERT will indicate those hypotheses needing testing in the next generation of trials in this disease.

## Methods and analysis

The CONVERT trial is an international, multicentre, prospective, non-blinded, superiority randomised controlled trial. The trial is sponsored by The Christie NHS Foundation Trust and coordinated by the Manchester Academic Health Science Centre Trial Co-ordination Unit (MAHSC-CTU) based at The Christie NHS Foundation Trust. The trial is registered on the International Standardised Randomised Controlled Trial Registry (ISRCTN91927162) and funded by Cancer Research UK's Clinical Trials Awards & Advisory Committee (CTAAC). The study is included in the NIHR Clinical Research Network portfolio (ID: 3823). The trial is conducted in accordance with the Declaration of Helsinki and Good Clinical Practice (GCP).

The primary research question is to establish whether the results of twice-daily chemo-RT for patients with LS-SCLC and good PS can be improved on, by delivering a higher dose of RT once-daily concurrently with chemotherapy. We will compare survival of patients treated with standard chemotherapy (cisplatin and etoposide) and either twice-daily RT or high dose once-daily RT.

The secondary research questions will compare the following factors between the groups receiving either once or twice-daily RT:

- Local progression-free survival
- Metastasis-free survival
- Acute and late toxicity based on the Common Terminology Criteria for Adverse Events V.3.0 (CTCAE V.3.0)^13^
- Chemotherapy dose intensity
- Radiotherapy dose intensity.

### Setting

Five hundred and forty-seven patients with a histological or cytological proven diagnosis of SCLC were recruited from 74 centres in eight countries between April 2008 and November 2013 (see online supplementary appendix 1 for details of recruiting centres).

**Figure 1:**
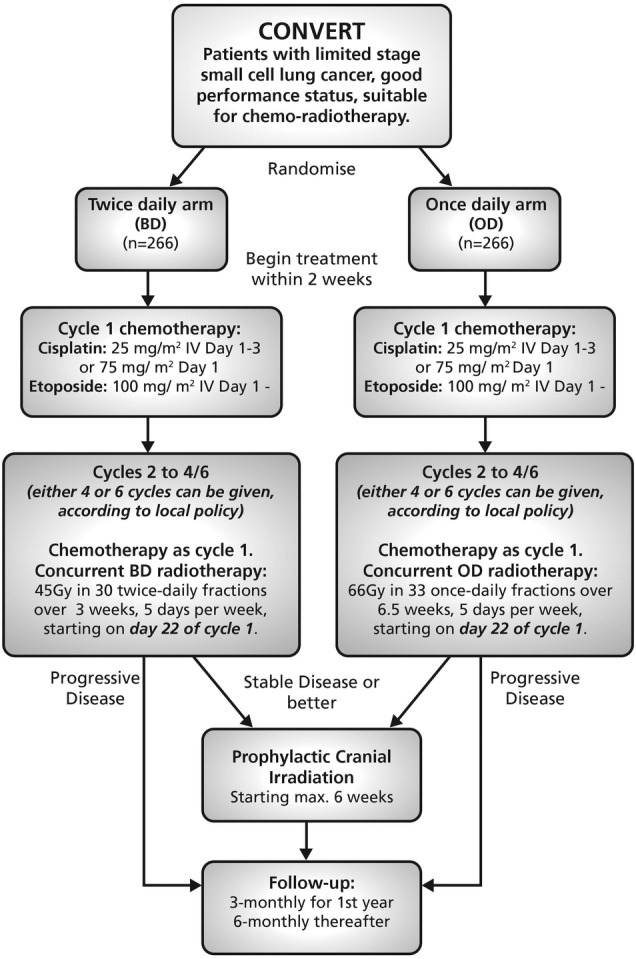
Trial schema/flowchart. BD, twice-daily; IV, intravenous; OD, once-daily.

Patients were randomised to receive either concurrent twice-daily RT (45 Gy in 30 twice-daily fractions over 3 weeks, 5 days per week, starting on day 22 of cycle 1) or concurrent once-daily RT (66 Gy in 33 daily fractions over 6.5 weeks, 5 days per week, starting on day 22 of cycle 1). Patients are followed up until death. The study flow diagram is shown in figure 1.

### Participant screening and selection

All patients with LS-SCLC with good PS and suitable for concurrent chemo-RT were identified as potential trial candidates. Eligible patients invited to participate were provided with a patient information sheet (see online supplementary appendix 2).

### Inclusion and exclusion criteria

Patients were eligible for the trial if all of the following criteria were met:

- Either sex, age ≥18 years.
- PS Eastern Cooperative Oncology Group grade 0–1. Patients with PS 2 whose general condition is explained by obstructive/bulky disease likely to improve after the first cycle of chemotherapy can be included at the discretion of the local investigator. Patients with PS 2 as a result of comorbid conditions will be excluded
- Histologically or cytologically confirmed SCLC
- No patients with mixed small-cell and non-small-cell histological features
- No history of previous malignancy in the past 5 years (except non-melanomatous skin or in situ cervix carcinoma). Patients with previous malignancies (except breast cancer) and in remission for at least 5 years can be included
- Limited stage disease (Veterans Administration Lung Cancer Study Group), that is, patients whose disease can be encompassed within a radical radiation portal
- No pleural or pericardial effusions proven to be malignant
- Radiotherapy target volume acceptable by the local radiotherapist
- Pulmonary function
  1. Forced expiratory volume in 1 s >1 L or 40% predicted value
  2. Transfer coefficient of the lung for carbon monoxide (KCO) >40% predicted
- Maximum of one of the following adverse biochemical factors:
  1. Serum alkaline phosphatase more than >1.5 times the upper limit of normal
  2. Serum sodium <lower limit of normal
  3. Serum lactate dehydrogenase >upper limit of normal
- Normal serum creatinine and calculated creatinine clearance ≥50 mL/min. If calculated creatinine clearance is <50 mL/mn according to the Cockroft and Gault formula, a glomerular filtration rate should be performed
- Adequate haematological function
  1. Neutrophils >1.5×10^9^/L
  2. Platelets >100×10^9^/L
- Adequate liver function: alanine transaminase and aspartate aminotransferase ≤2.5×upper limit normal
- No other previous or concomitant illness or treatment that, in the opinion of the clinician, will interfere with the trial treatments or comparisons
- No prior surgical resection of the primary tumour, no prior RT for lung cancer.
- Considered fit to receive any of the trial regimens
- Female patients must satisfy the investigator that they are not pregnant, or are not of childbearing potential, or are using adequate contraception. Men must also use adequate contraception
- Patients must not be breastfeeding
- Patient has read the patient information sheet and has signed the consent form
- Patients available for follow-up

### Informed consent

Eligibility to participate was confirmed by a clinician prior to consent being taken. Patients were given at least 24 h to consider the patient information sheet and time to ask questions prior to written informed consent being taken by a trial doctor. The consent form can be viewed in online supplementary appendix 3.

### Randomisation

Randomisation was administered centrally by the MAHSC-CTU. Patients were randomised on a 1:1 basis to one of the two treatment arms. The allocation method used was minimisation with a random element. Randomisation was implemented via a bespoke computer application at the randomisation centre. The factors controlled for in the allocation were institution, planned number of cycles (4 or 6) and PS (0/1 or 2). Randomisation was only performed after confirmation that the patient was eligible (including recording of LDH, sodium and alkaline phosphatase results) and that the patient had signed consent. Randomisation can be undertaken by telephone or fax. The system used did not permit any editing of fields by users after arm allocation had been performed.

### Standard care

Concurrent chemo-RT is the standard of care in LS-SCLC and good PS. The combination of cisplatin and etoposide is the standard chemotherapy treatment delivered concurrently with RT in this group of patients. One of the accepted international standard RT regimes is 45 Gy in 30 fractions delivered twice-daily, the control arm of the CONVERT trial.^3^ However, in reality, RT regimes differ widely between institutions and twice-daily RT has not been adopted widely mainly due to logistical issues.^12^

### Interventions

In both arms, three-dimensional conformal RT was used and 4–10 MV photons emitted from linear accelerators. Thoracic RT was started on cycle 1, day 22, concurrently with the second cycle of chemotherapy, where possible. Intensity modulated RT and positron emission tomography-CT planning were permitted but not mandated. The full trial-specific procedure for RT can be found in online supplementary appendix 4, including the definition of the Planning Target Volume. However, it is important to note that, in this trial, clinically uninvolved lymph node stations were not irradiated.^14^

Patients randomised to the twice-daily thoracic RT arm received 45 Gy in 30 twice-daily fractions over a period of 19 days, five consecutive days a week. The optimal overall treatment time was 19 days. The inter-fraction interval was 6–8 h. Concurrent chemotherapy was administered during the intervals between the two daily RT fractions.

Patients randomised to the high dose once-daily thoracic RT arm received 66 Gy in 33 daily fractions over a period of 45 days, five consecutive days a week. The optimal overall treatment time was 45 days.

Patients received cisplatin and etoposide, for four to six cycles, every 3 weeks, in both arms. Centres were given the choice to stop chemotherapy after four cycles or to continue it up to six cycles. Centres that decided to give six cycles were asked to continue doing so for all patients entered in the trial (unless it was decided that it was not in the patients’ best interest to receive cycle 5 and 6 or due to patient's choice). The two regimens of chemotherapy permitted were: (1) etoposide 100 mg/m^2^ intravenous day 1–3 and cisplatin 75 mg/m^2^ intravenous day 1, or (2) etoposide 100 mg/m^2^ intravenous day 1–3 and cisplatin 25 mg/m^2^ intravenous day 1–3. The use of granulocyte-colony stimulating factor during chemotherapy was permitted.^15^

No later than 6 weeks after the last cycle of chemotherapy, patients without evidence of progressive disease on chest X-ray or CT scan and with no clinical evidence of brain metastases were given prophylactic cranial irradiation.

### Translational research

Progress in treatment of SCLC has been hampered by limited understanding of the molecular biology of this disease. It is usually diagnosed on a small biopsy specimen or fine needle aspirate insufficient for detailed molecular studies. Consequently, existing SCLC tumour banks include relatively small series (<100 patients) of samples collected over many years from patients who are heterogeneous with respect to stage and treatment received. The CONVERT trial provides a unique opportunity to prospectively collect a large number of biospecimens from patients of uniform (limited) stage, who are exposed to the same chemotherapy, treated with one of two RT schedules and for whom there will be robust clinical outcome data. Although it will still be problematic to obtain large tumour biopsy specimens for many patients, advances in genomic and proteomic technology will enable studies to be performed on blood/serum samples in addition to small biopsy specimens.

All patients were asked to consent for an optional collection of tumour samples (paraffin embedded*)* and blood samples as part of the trial. Blood samples (for genomic and proteomic analysis) were collected at three time points: at baseline prior to any treatment, on day 22 of treatment and on completion of treatment. The sample collection schedule is shown in table 1.

**Table 1:** Sample collection schedule

	Baseline	Day 22	End of treatment
Tissue block			
Serum	✓	✓	✓
Plasma	✓	✓	✓
Whole blood	✓		
CTC	✓		

CTC, circulating tumour cells.

### Data collection and management

Participating centres completed the following case report forms (CRFs):

- Eligibility checklist prior to or at the time of randomisation
- Pre-treatment and tumour assessment at baseline prior to cycle 1
- Treatment forms on day 1 of each chemotherapy cycle (cycles 1–4 or 6). The data collected included PS, protocol treatments received, toxicity and reasons for reduction/delay/omission of treatment
- Toxicity forms at the end of each cycle given, prior to next cycle and 30 days after completion of the last cycle of chemotherapy
- Radiotherapy worksheet during and after completion of RT
- Post treatment form 30 days after the last cycle of chemotherapy
- Follow-up forms at each follow-up visit starting at 3 months postcycle 4 (or 6) visit (continuing 3 monthly until 12 months and then 6 monthly thereafter)
- Serious adverse event (SAE) forms used to report all SAEs
- Progression/relapse/death forms to report the patient status.

Copies of all CRFs continue to be returned to the trials centre for statistical analysis. All forms are tracked and entered into a study-defined database for which consistency checking is programmed in. Data managers check for missing and invalid data using SQL queries and statistical programmes. Any queries raised are returned to the centres for correction or clarification.

On completion of the study, the data will be written onto compact disk and archived in a safe and secure location within the MAHSC-CTU. Paper copies of the CRF's will be retained at sites for at least 15 years following the last, patient entered or, if all are deceased may be archived off site. All paper data will be destroyed after 15 years on the approval of the chief investigator.

The trials centre staff are in regular contact with local centre personnel to check on progress and to help with any queries that may arise. Incoming forms are checked for completeness, consistency, timeliness and compliance with the protocol.

### Sample size calculation

It is considered that a survival benefit of 12% at 2 years (in favour of once-daily RT) would be clinically significant. Using Freedman's sample size calculation based on a two-arm trial, with a 5% significance level, two-sided test, 80% power and HR of 0.70, a 12% overall survival benefit at 2 years from 44% with the control arm to 56% in the experimental arm, required a total of 506 patients. The number of deaths required is 247. An additional 5% was added to allow for ineligible patients, giving a total of 532 patients required. An additional 15 patients were recruited (total of 547) to replace patients either randomised in error or for whom we were never able to obtain data.

### Statistical analysis plan

The analysis will be based on intention-to-treat principles (ITT). Patient data will be grouped by treatment arm according to the treatment assignments made via the MAHSC-CTU randomisation line. The primary data analysis is planned for January 2015.

The stratification factors include centre, PS (0–1 vs 2) and biochemical factors (eg, LDH, sodium, alkaline phosphatase). Comparison of data by centre will be scrutinised to identify any data inconsistencies, it will also be used to identify those centres planning to give either four or six cycles of chemotherapy. Analysis will be carried out to identify any differences between the two schedules. Other factors have been identified in previous studies to be prognostic factors and will be used to calculate the Manchester score, which gives three groupings of good, intermediate and poor prognosis; these scores will be used to compare OS and response rate.

The only planned interim analyses have been performed for the Independent Data Monitoring Committee (IDMC). Reports have been submitted to the IDMC on an annual basis starting 12 months after the first patient was randomised.

The following are the qualifications for analysis of time-to-event efficacy parameters:

- All randomised patients will be included in the analysis of overall survival (OS) and local progression free survival (ITT).

OS is the time between date of randomisation and date of death of any cause. Survivors will be censored on the last date known to be alive. Local progression-free survival (local control) will be calculated from the date of randomisation to the date of first clinical evidence of progressive disease at the primary site, or death. Kaplan-Meier curves will be drawn for each treatment group. Overall survival and local progression-free survival will be compared using the Mantel-Cox version of the log rank test.

- All randomised patients treated with at least one study dose of cisplatin and etoposide will be included in the comparison of proportions of grade 3 and 4 toxicities.

Toxicity will be assessed according to NCI Common Terminology Criteria for Adverse Events V.3.0. The proportion of patients experiencing a grade of 3 or above acute toxicity, including acute and/or late radiation morbidity, will be compared between the treatment groups using χ^2^ and Fisher Exact tests. Acute toxicity will be defined as toxicities occurring from commencement to 3 months after completion of treatment; late toxicity will be defined as toxicities occurring between 3 months and 2 years after completion of treatment.

All patients treated with at least one dose of cisplatin and etoposide will be evaluated for tumour response and included in the analysis of tumour response rates. Response will be assessed according to Response Evaluation Criteria In Solid Tumours (RECIST) criteria. The proportion of patients in each treatment group whose best response (up to approximately 28 days postcycle 4 or, if stopped prior to cycle 4, approximately 28 days after last chemotherapy cycle given) from randomisation is complete or partial will be compared using χ^2^ and Fisher Exact tests.

Safety analyses will be performed for all randomised patients treated with at least one dose of chemotherapy. Adverse effects will be summarised and compared between the two arms.

The relative chemotherapy and RT dose intensity (RDI) will be summarised by calculating the median, SD, IQR and range for patients in each randomised treatment group. The RDI will be compared between the treatment groups by using the Wilcoxon Rank Sum test.

A detailed description of patient disposition will include a summary of the following:

- All patients entered and enrolled: overall, by treatment arm and by country
- Reasons for patients entered but not enrolled
- All enrolled patients treated with study drug, by treatment arm
- Reasons patients enrolled but not treated with study drug
- Reasons patients discontinued study drug treatment
- All important protocol violations.

### Changes to the protocol after the start of the trial

The trial details documented here are consistent with CONVERT trial protocol V3 (dated: 10 June 2008). There were no significant changes to the protocol after the start of the trial, only minor administrative amendments and clarifications have been made during the course of the trial.

### End of the trial

The trial will end once all 547 patients recruited have died or completed 5 years of trial follow-up (whichever is sooner).

## Ethics and dissemination

### Radiotherapy quality assurance

The trial is subject to a RT quality assurance programme, managed by the National Cancer Research Institute Radiotherapy Trials Quality Assurance Team (RTTQA). Participating centres were provided with RT planning guidelines, including an atlas of organ at risk delineation, and had to pass an initial assessment before patients could be randomised into the trial, and there were further assessments afterwards.

The initial assessment consisted of:

- Completion and return of a questionnaire detailing the RT facilities available to the centre
- Return of two RT treatment plans for a patient with limited-stage SCLC, previously treated in the centre with radical intent, who satisfied the eligibility criteria for CONVERT and had been replanned according to the CONVERT protocol for each treatment arm: 66 Gy in 33 daily fractions once-daily and 45 Gy in 30 fractions twice-daily

During the trial, plans were randomly requested from each centre as part of the continuing quality assurance programme and feedback was provided in case of protocol deviations. Participating centres had to agree to address uncertainties revealed by the quality assurance programme.^16^

### Safety reporting

Data were collected at each trial visit regarding any SAEs (as defined by Good Clinical Practice Guidelines). All SAEs causally related to either chemotherapy or RT treatment were reported to the MAHSC-CTU and followed until they resolved or stabilised. Late radiation toxicities continue to be recorded at each follow-up visit (according to the CTCAE V.3.0 grading system).

### Early stopping of trial treatment

Protocol treatment may be stopped in the following instances:

- There is evidence of progressive disease according to RECIST criteria on CT scan (see online supplementary appendix 4)
- Unacceptable toxicity
- Early toxicity assessment (after 20 patients have completed treatment in each arm), which, for safety reasons, will be carried out. Data will be reviewed by the Trial Management Group (TMG)
- Intercurrent illness exists that, in the clinician's opinion, would require discontinuation of protocol therapy.
- Subsequent histological/cytological review is contrary to the original diagnosis
- Patient's request.

### Trial monitoring and oversight

No formal on site data monitoring activities were performed as part of the CONVERT trial.

Data are reviewed annually by an IDMC, consisting of three clinicians not entering patients into the trial, and an independent statistician. Throughout the duration of the trial, the IDMC has recommended whether the accumulated data from the trial, together with results from other relevant trials, justified continuing recruitment of further patients. The IDMC has made confidential recommendations to the Trial Steering Committee (TSC).

The role of the TSC has been to act on behalf of the funder, to provide overall supervision for the trial, to ensure that it is conducted in accordance with GCP and to provide advice through its independent Chairman. This independent committee reviews the recommendations from the IDMC and decides on continuing or stopping the trial or modifying the protocol. The Trial Management Group coordinates and manages the trial's day-to-day activities. The TMG is comprised of health professionals and members of the direct study team.

### Dissemination

Data from all centres will be analysed together and published as soon as possible. Individual participants may not publish data concerning their patients that are directly relevant to questions posed by the trial until the TMG has published its report. The TMG will have access to the final data set, form the basis of the Writing Committee and advise on the nature of publications. The trial will be publicised at regional and national conferences. The final results will be presented at scientific meetings and published in a peer-reviewed journal (authorship will be according to the journal's guidelines).
